# Phagomania

**Published:** 1881-10-20

**Authors:** L. G. Hardman

**Affiliations:** Georgia


### PHAGOMANIA.

BY L. G. HARDMAN, M. D., OF GEORGIA.

The description of this condition will give a sufficient idea of the definition of the term heading this article. It is, however, derived from two words, meaning hunger and madness; therefore, this patient, you will discover, is roaming about with eating-insanity.

Mr. B., aged 23 years, farmer by occupation; stout, healthy, robust boy up to four years of age, but during this time was known to have a voracious appetite from childhood, and this great desire for food seemed to increase, and with it insanity. There were times or paroxysms in which he commenced to steal food, and this continued to increase with the insanity, which caused him to make attempts to commit suicide and homicide. He at one time made an incision into his larynx, at which time I was summoned to see him, and on arrival found a considerable wound, from which he recovered.

During these paroxysms he sometimes goes about over the country stealing food. Thus you see the appellation applied to this disease or condition expresses the true nature of the case.

His immediate relation on the mother's side was subject to insanity, and on the father's side to dispepsia; so you discover we have here the disturbance in both the nervous and digestive systems. He at one time took but little food, and during this time the insanity was not so violent. At this time he was very much emaciated, but shortly his voracious appetite returned and with it an increase of the insanity and the stealing propensity, food being the thing always stolen. When he could get food he would eat until pains in his bowels commenced, and then toss himself on the floor until relieved by vomiting, after which he would repeat the same thing immediately, if he could get the food.

During the paroxysm of insanity his great animosity is at his mother and sisters, whom he would murder if left alone.

His father was thought by the people to be a man of good mind, and doing well financially, until recently he left his family and went West. Since which time it has been discovered that his mind is not right.

There are other children in the family who have not strong minds, but in whom, as yet, insanity has not developed.

The name above given to this affliction I believe has never been used, but it expresses a condition no other word could. Kleptomania refers to stealing generally, but we have in this stealing a propensity for a particular article.

